# Supplementary material for: A Global Screen for Assembly State Changes of the Mitotic Proteome by SEC-SWATH-MS
Source: Cell Syst. 2020 Feb 26;10(2):133–155.e6. doi: 10.1016/j.cels.2020.01.001 (PMC7042714; doi:10.1016/j.cels.2020.01.001)

P06733 | ENOA_HUMAN | ENO1 ENO1L1 MBPB1 MPB1
Monomer MW [kDa]: 47.169 Monomer expected elution fraction: 47

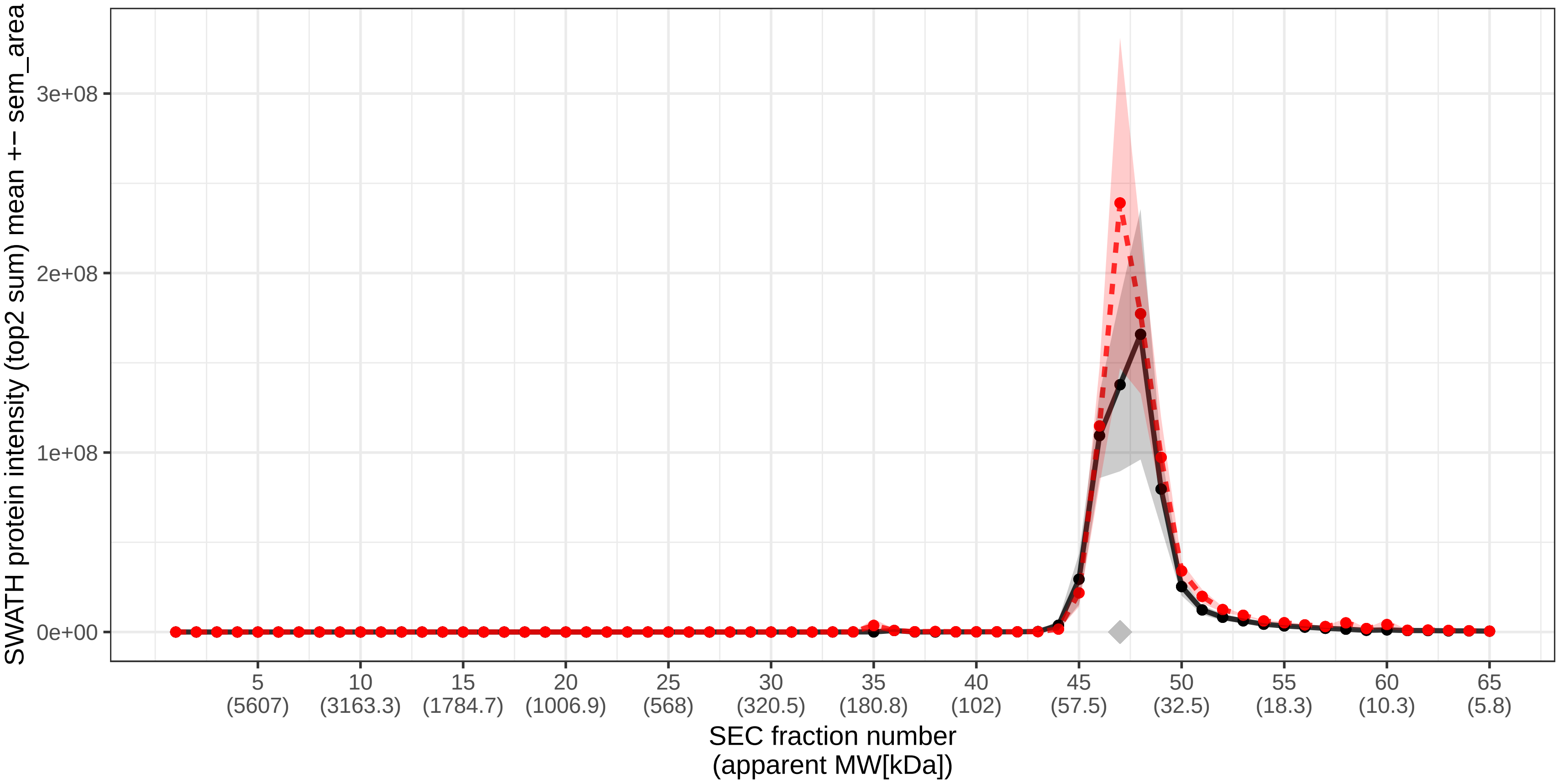

Supplement: Data S1. SEC-SWATH-MS Protein Chromatograms, Related to Figure 1 [file mmc6.zip › SECchrom_P06733_ENOA_HUMAN_ENO1_ENO1L1_MBPB1_MPB1.pdf]
